# The SAGES masters program: top 10 seminal articles for laparoscopic fundoplication

**DOI:** 10.1007/s00464-025-12141-1

**Published:** 2025-09-08

**Authors:** Daphne Remulla, Shelley Ivary, Emanuele Lo Menzo, Ruchir Puri, Jon C. Gould, Lucas R. Beffa

**Affiliations:** 1 Department of General Surgery, Digestive Disease Institute, Cleveland Clinic Foundation, Cleveland, OH USA; 2 Cleveland Clinic Floyd D. Loop Alumni Library, Cleveland Clinic Foundation, Cleveland, OH USA; 3 Ellen Leifer Shulman and Steven Shulman Digestive Disease Center, The Bariatric and Metabolic Institute, Cleveland Clinic Florida, Weston, FL USA; 4 Department of Surgery, University of Florida College of Medicine Jacksonville, Jacksonville, FL USA; 5 Division of General Surgery, Department of Surgery, Medical College of Wisconsin, Milwaukee, WI USA

**Keywords:** SAGES masters program, Laparoscopic fundoplication, Gastroesophageal reflux disease, Top 10, Surgical education

## Abstract

**Background:**

As one of the eight clinical pathways of the Society of American Gastrointestinal and Endoscopic Surgeons (SAGES) Masters Program, the Foregut Pathway includes laparoscopic fundoplication as an anchoring procedure. In this article, the SAGES Foregut Committee presents focused summaries of the top 10 seminal articles selected for laparoscopic fundoplication which surgeons performing this procedure should be familiar with.

**Methods:**

A systematic literature search was performed by a research librarian using Web of Science and Google Scholar. The top 30 papers with the highest citation index were extracted, reviewed, and ranked according to importance by members of the SAGES Foregut Committee. Additional articles not identified in the literature search were included if deemed impactful by expert consensus. The top 10 ranked articles were then summarized, with emphasis on relevance and impact in the field, findings, strengths and limitations, and conclusions.

**Results:**

The top 10 seminal articles selected for the laparoscopic fundoplication anchoring procedure include clinical practice guidelines, randomized controlled trials comparing surgical techniques and medical versus surgical therapy, systematic reviews and meta-analyses evaluating outcomes, and foundational papers establishing technical principles. These articles address patient selection, operative techniques, clinical outcomes, and evidence-based recommendations for the surgical management of gastroesophageal reflux disease.

**Conclusions:**

The top 10 seminal articles selected for laparoscopic fundoplication are considered by the SAGES Foregut Committee to be foundational in supporting surgeon progression to mastery within the Foregut Pathway. These papers establish the fundamental principles and evidence that guide the current practice of laparoscopic fundoplication.

**Supplementary Information:**

The online version contains supplementary material available at 10.1007/s00464-025-12141-1.

The field of surgery is continuously expanding, with advancements in surgical techniques, new technologies, and evolving evidence-based practices. This continued expansion in knowledge and technology represents a significant challenge for practicing surgeons who must not only stay up to date with current literature and evidence to provide optimal patient care, but also master technical skills and educate both patients and trainees. To address the lifelong learning needs of practicing surgeons after the completion of training, the Society of American Gastrointestinal and Endoscopic Surgeons (SAGES) developed a Masters Program. This program offers educational content across eight clinical pathways relevant to gastrointestinal and endoscopic surgery: Acute Care, Bariatric, Biliary, Colorectal, Foregut, Hernia, Robotic Surgery, and Flexible Endoscopy. Each clinical pathway contains three progressive skill levels: competency, proficiency, and mastery. Each level features specific core or "anchoring procedures" that represent a longitudinal progression of technical complexity and serve as the basis for training, assessment, and coaching.

As part of the Masters curriculum, seminal articles are identified for each anchoring procedure. Seminal articles represent key literature that is considered relevant, impactful, and foundational to practicing surgeons. These papers capture research studies and evidence-based guidelines on the surgical techniques, clinical outcomes, disease pathophysiology, and technological advances that every surgeon performing the associated anchoring procedure should know. This curated list of articles serves as an invaluable resource for surgeons to improve their knowledge and inform best practices as they progress through the clinical pathways and levels of expertise. In this article, we present the Top 10 seminal articles for laparoscopic fundoplication as part of the Masters’ Foregut pathway. This initiative supports SAGES’ goal of promoting lifelong learning and continuous improvement among practicing gastrointestinal and endoscopic surgeons performing these operations.

## Methods

**Fig. 1:**
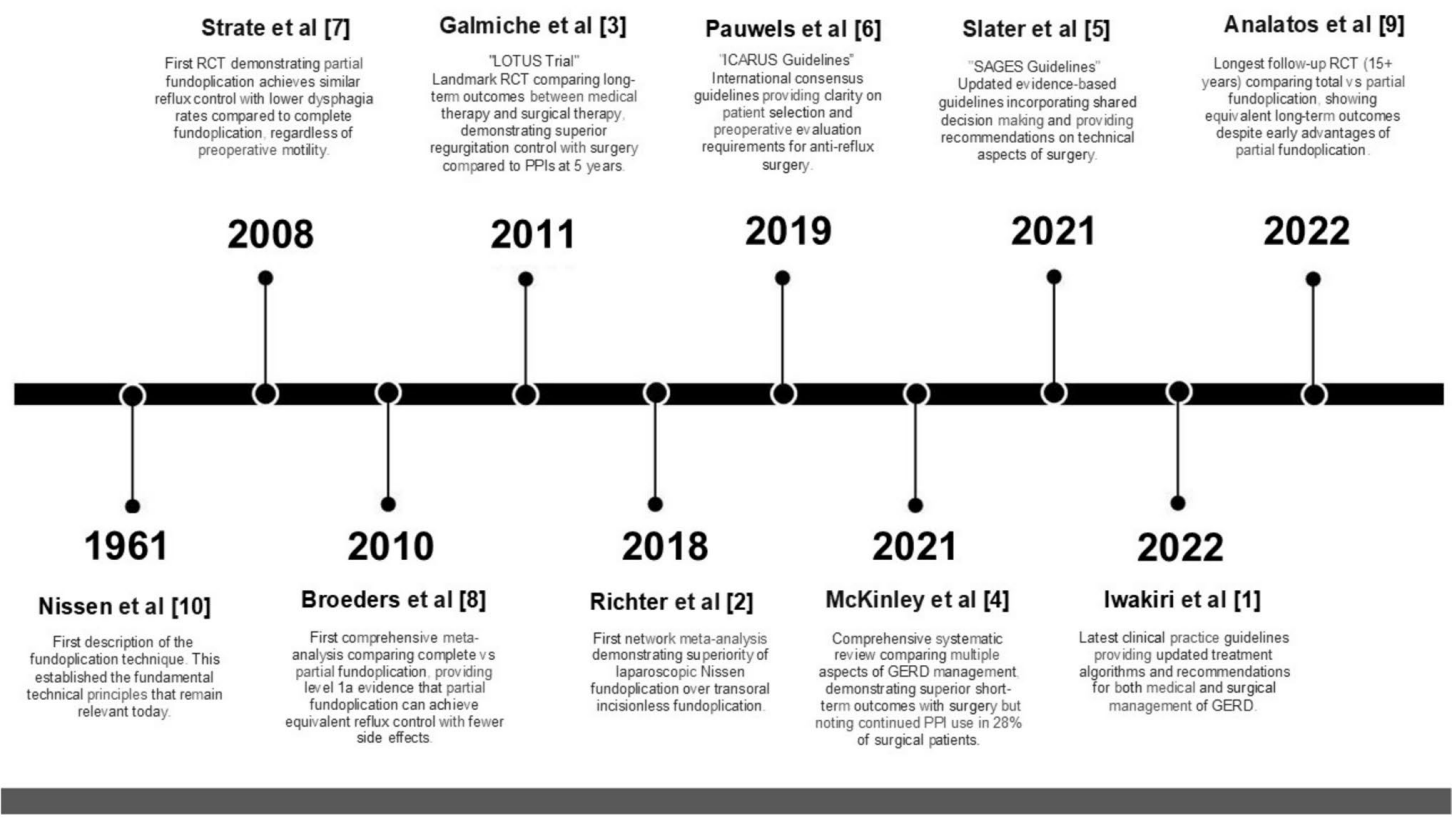
Publication timeline depicting top 10 articles for laparoscopic fundoplication

SAGES Masters Program used a previously defined, standardized methodology to identify seminal articles for each anchoring pathway [1]. In this method, a systematic literature search was performed by a research librarian using Google Scholar and the Web of Science platform [Supplementary 1]. For each identified paper, a citation index (CI) was calculated using the equation: CI = number of citations/years since publication. This search was initially performed by the SAGES Foregut Committee in 2018 and updated in February 2024. The results of the search were manually reviewed to ensure relevance to the anchoring procedure, laparoscopic fundoplication. All articles were ranked by CI, and the top 30 articles with the highest CI were reviewed by members of the SAGES Foregut Committee. Reviewers were permitted to suggest additional manuscripts not identified in the search if deemed impactful to the teaching, training, and/or safe adoption of the anchoring procedure. Foregut Committee members ranked the 30 identified articles by importance generating a consensus determination of the top 10 seminal articles in laparoscopic fundoplication. The top 10 articles were then divided among Foregut Committee members for individual review to generate written synopses of their assigned articles. Emphasis was placed in each summary on the following key questions: (1) Why is this a top 10 article? (2) What is unique about this paper? (3) Why is it important to read this paper before performing the relevant procedure? (4) What has been the impact of this paper in the field? (5) What are the study findings? (6) What are the strengths and limitations of paper, and (7) What are the conclusions of this article? Summaries were compiled and reviewed by Foregut Committee members through iterative group discussions during which members offered feedback, suggested changes, and provided revisions (see Table 1 and Fig. 1).

**Table 1:** Citation indices for the top 10 articles for the laparoscopic fundoplication anchoring procedure

Rank	First author	Year	Web of science citations (*n*)	Web of science CI	Google Scholar citations (*n*)	Google Scholar CI
1	Iwakiri	2022	50	50.0	52	52.0
2	Richter	2018	74	14.8	136	27.2
3	Galmiche	2011	325	27.1	322	26.8
4	McKinley	2021	43	21.5	30	15
5	Slater	2021	16	8.0	42	21.0
6	Pauwels	2019	72	18.0	66	16.5
7	Strate	2008	74	14.8	257	17.1
8	Broeders	2010	176	13.5	184	14.2
9	Analatos	2022	12	12.0	13	13
10	Nissen	1961	Committee Consensus			

*CI = citation index = (number of citations/years since publication)

## Results

1. Iwawkiri et al. Evidence-based clinical practice guidelines for gastroesophageal reflux disease 2021 [2].

In this manuscript, Iwakiri et al. provide updated clinical practice guidelines for the medical and surgical treatment of gastroesophageal reflux disease (GERD) from the Japanese Society of Gastroenterology (JSPE). The guideline committee stratified questions into clinical questions (CQ), background questions (BQ), and future research questions (FRQ) and then assessed the quality of evidence for each question using the Grading of Recommendations Assessment, Development and Evaluation (GRADE) system. They also provided recommendations were indicated as either “strong recommendation” or “weak recommendation” based on a modified Delphi process wherein > 70% of votes were agreed upon by the JSPE committee members.

Key findings:

- The development of novel treatment algorithms that classify GERD into reflux esophagitis (RE) and non-erosive disease (NERD).
- Updated recommendations for
  o Vonoprazan (a competitive potassium-competitive acid blocker, P-CAB) as first-line treatment and as maintenance therapy for severe RE. (weak recommendation)
  o Either a PPI or vonoprazan as first-line for mild RE (strong recommendation)
- All recommendations for surgical therapy are weakly recommended for drug-resistant RE and NERD patients due to moderate/low quality evidence.

This clinical guideline published in 2021 provides relevant evidence-based decision-making and highlights the importance of medical therapy while also recognizing its limitations and when surgical anti-reflux procedures are indicated. This paper warrants inclusion as a top 10 article as it represents the most up-to-date evidence-based guidelines for GERD management developed through rigorous international consensus. Its major strength is its methodology using GRADE assessments and expert consensus through a Delphi process. Key limitations include the reliance on moderate to low quality evidence for surgical recommendations and lack of high-quality data to support strong recommendations in many areas. Nevertheless, these guidelines are highly relevant for practicing surgeons to understand proper patient selection and indications for anti-reflux surgery and have thus rightfully been selected as a seminal foregut article by the SAGES Foregut Committee.

2. Richter et al. Efficacy of Laparoscopic Nissen Fundoplication vs Transoral Incisionless Fundoplication or Proton Pump Inhibitors in Patients With Gastroesophageal Reflux Disease: A Systematic Review and Network Meta-analysis [3].

This systematic review and network meta-analysis compares outcomes between laparoscopic Nissen fundoplication (LNF) and transoral incisionless fundoplication (TIF) in the absence of a direct comparative study at the time of publication. To do so, the authors identified and included randomized controlled trials evaluating the efficacy of TIF or LNF to medical therapy or sham procedures for the treatment of GERD and then used network meta-analysis methodology to enable indirect comparison of the two surgical approaches. After applying the inclusion criteria, seven RCTs were included for analysis. The primary outcome of this analysis was a decrease in proportion of 24-h time spent at pH < 4 and augmentation of lower esophageal sphincter pressure (LESP). Secondary outcomes evaluated included health-related quality of life (HRQOL) and serious adverse events. The seven included RCTs were assessed for risk of bias and heterogeneity/reporting bias, and the overall quality of the trials and evidence were evaluated using the GRADE method.

Key findings:

- LNF is superior to TIF with decreased percentage of time of pH < 4 (OR 0.08; 95% CrI 0.02–0.36)
- TIF showed trend toward improved quality of life; however, this difference was confounded by shorter follow-up duration in the TIF group (regression coefficient 0.83; 95% CI 0.61–1.04; *p* > 0.001).
- Other trends that did not reach statistical significance.
  o TIF was superior to LNF with improved quality of life.
  o LNF was superior to TIF due to higher LESP and decreased esophagitis.

This meta-analysis deserves inclusion as a seminal article for providing the first comprehensive comparative analysis between two distinct endoscopic and laparoscopic anti-reflux procedures for GERD and addressing a critical knowledge gap in the absence of direct head-to-head trials. The data presented here support LNF as a superior anti-reflux procedure compared to TIF on multiple key objective measures including time of pH exposure and LESP. Strengths include its sophisticated use of network meta-analysis, which allows for comparative effectiveness of interventions that may or may not have been compared directly against each other through a common comparator. Several important limitations of this trial to note include the heterogeneity of techniques used, lack of blinding, lack of standardization, and very low to moderate quality of the RCTs included in this analysis. By providing data surrounding a highly relevant and clinically meaningful question, this study by Richter and colleagues warrants inclusion as a seminal article.

3. Galmiche et al. Laparoscopic Antireflux Surgery vs Esomeprazole Treatment for Chronic GERD—The LOTUS Randomized Clinical Trial [4].

The LOTUS (Long-Term Usage of Esomeprazole vs Surgery for Treatment of Chronic GERD) trial was a 5-year exploratory, randomized, open, parallel trial from 11 European teaching hospitals. Two hundred sixty-six patients were randomized to receive medical treatment with esomeprazole (20 to 40 mg/d), and 248 patients were randomized to Laparoscopic Anti-Reflux surgery (LARS). The primary outcome measure of this trial was the time to treatment failure, which was defined as inadequate symptom control after dose adjustment in the esomeprazole group and the need for acid suppression in the LARS group. Follow-up included a clinic visit every 6 months after randomization. Upper endoscopy was scheduled at 1, 3, and 5 years, and distal esophageal acid exposure was assessed at baseline, 6 months, and 5 years respectively.

Key findings:

- Treatment failure occurred in 33/180 surgical vs 19/192 medical patients at 5 years.
- Superior regurgitation control with surgery (2% vs 13%, *p* < 0.001)
- Higher rates in surgical group of:
  o Dysphagia (11% vs 5%, *p* = 0.001)
  o Bloating (40% vs 28%, *p* = 0.001)
  o Flatulence (57% vs 40%, *p* = 0.001)
- Similar rates between groups of
  o Heartburn (8% vs 16%, *p* = 0.14)
  o Epigastric pain (18% vs 18%, *p* = 0.55)
  o Lower esophageal acid exposure with surgery (0.7% vs 1.9%)
- 3% surgical morbidity with no mortality

As one of the first contemporary RCTs to compare medical and surgical therapy for chronic GERD, the LOTUS trial found that patients maintain and achieve remission at five years with either esomeprazole or LARS, with surgery offering superior regurgitation control and higher rates of mechanical side effects. Key strengths include the large, randomized design, 5-year follow-up with > 70% retention, and multi-institutional participation. Major limitations include enrollment of only PPI responders and lack of standardized surgical technique description. Another limitation of this study is that esomeprazole is more efficacious than other PPIs; therefore, these results may not be applicable to other PPIs which are metabolized via different pathways. This landmark RCT provided early high-quality evidence comparing long-term outcomes between medical and surgical therapy, demonstrating that both approaches can achieve and maintain remission at 5 years.

4. McKinley et al. Surgical treatment of GERD: systematic review and meta-analysis [5].

This systematic review and meta-analysis by McKiney et al. evaluates outcomes of anti-reflux surgery versus medical management of GERD in adults and children, robotic versus laparoscopic fundoplication, complete versus partial fundoplication, and minimal versus maximal dissection in pediatric patients. Using three databases, a total of 105 studies published between 2004 and 2019 were analyzed and assessed for quality and reliability using the Cochrane Risk of Bias Took and Newcastle Ottowa Scale.

Key findings:

- Antireflux surgery was associated with superior short-term quality of life compared to PPI therapy; however, this difference is not seen on long-term (> 5 year) follow-up.
- A high percentage (28%) of operative patients continue PPI treatment postoperatively.
- The robotic approach was more costly and symptom control, and complication rates were similar compared to laparoscopic fundoplication.
- Partial fundoplication was associated with higher rates of prolonged PPI usage but no difference in long-term symptom control or long-term dysphagia.
- In pediatric patients, minimal dissection during fundoplication was associated with lower reoperation compared to maximal dissection.

The paper warrants inclusion as a top 10 article because it represents a comprehensive, methodological, and rigorous analysis that addresses multiple key controversies in the surgical management of GERD, including patient selection between medical and surgical therapy, the choice of surgical approach (robotic vs laparoscopic), technical decisions regarding complete vs partial fundoplication, and the recommended extent of dissection in pediatric cases. The strengths of this analysis include the large number of included studies identified by a systematic and comprehensive literature review, multiple clinically relevant outcomes and patient populations analyzed, and rigorous methodology. Limitations include limited long-term comparative data significant heterogeneity in surgical techniques and outcome definitions between studies and the inclusion of studies with high or uncertain risk of bias. The authors concluded that surgical therapy appears to provide superior short-term symptom control and higher quality of life over medical therapy in appropriately selected patients, with the caveat that a proportion of these patients will continue PPI treatment. This meta-analysis has become a seminal paper by establishing evidence-based practices across multiple key questions in GERD surgery using the highest level of evidence synthesis.

5. Slater et al. [6]. SAGES guidelines for the surgical treatment of gastroesophageal reflux (GERD) [6].

In this paper, the SAGES guidelines are updated from 2010, incorporating shared decision-making and patient values in the management of GERD. A multidisciplinary panel was convened that included adult and pediatric surgeons, gastroenterologists, a methodologist with guideline development expertise, and the SAGES Guidelines Committee Fellow. The panel formulated five key questions and graded the recommendations using the GRADE approach [7–9] and the GRADE guideline development tool. The panel then anonymously voted on the final recommendations where at least an 80% agreement was mandatory.

Key findings:

- Adults with chronic or chronic refractory GERD may benefit from surgical fundoplication over medical management. For pediatric patients, there is evidence to suggest that the fundoplication may be beneficial in children on 10 year or longer follow-up [10].
- Either robotic or laparoscopic fundoplication is recommended for both children and adults based on shared decision-making.
- Either partial or complete fundoplication recommended for adults, with choice based on patient preference for reflux control versus dysphagia. In children without a large hiatal hernia, either a partial or complete fundoplication can be utilized.
- Either division or no division of the short gastric vessels are acceptable for adults, with choice based on patient preference for superior reflux control (division) versus decreased long-term risk of gas bloat (no division).
- Minimal dissection should be used in pediatric patients due to decreased endoscopic dilation, reoperation for wrap failure, readmission for respiratory cause, and weight gain.
- All recommendations were conditional due to low/moderate certainty of evidence.

These guidelines provide comprehensive, evidence-based recommendations focused on surgical decision-making and emphasize individualization based on patient values. The guidelines are unique in addressing technical aspects of surgery while incorporating shared decision-making principles. Though several guidelines have been published related to GERD, the strengths of the SAGES guidelines for GERD are its methodology, focus on patient-centered outcomes, and comprehensive scope. Key limitations include low certainty evidence for all recommendations and limited long-term outcome data without bias. This work is included as a seminal article as the guidelines presented represent foundational, evidence-based frameworks that currently shape standards of care, influence research directions, and drive advancements in the field.

6. Pauwels et al. How to select patients for anti-reflux Surgery? The ICARUS guidelines international consensus regarding preoperative examinations and clinical characteristics assessment to select adult patients for anti-reflux surgery [11].

The ICARUS guidelines provide clarity for clinical decision-making and diagnostic requirements for anti-reflux operations through an international expert consensus which previously remained controversial. In this article, the International Society for Diseases of the Esophagus (ISDE) assembled 35 international experts and developed 37 statements through a Delphi process. The statements were graded based on consensus (Six points Likert scale) from A + agree strongly to D + strongly disagree and strength of the statement (GRADE system). Consensus was defined as 80% agreement among the group.

Key findings:

- Criteria for good surgical candidates
  o Patients with typical symptoms of heartburn responding to PPIs (Grade A).
  o Patients with GERD symptoms and endoscopic evidence of hiatal hernia (HH), esophagitis (LA grade B or higher) off PPI, or biopsy confirmed Barrett’s (Grade B).
  o Patients with small to large sliding hiatal hernias or paraesophageal hernias (Grade B/C).
- Criteria for poor surgical candidates
  o Patients with functional heartburn (Rome II/IV criteria) with no symptom association (Grade B).
  o Patients with eosinophilic esophagitis (EOO) (Grade C).
  o Patients with normal acid exposure on pH (± impedance) monitoring (Grade B).
- Preoperative work-up recommendations.
  o Mandatory: Upper endoscopy within 1 year (Grade B), esophageal manometry for patient selection (Grade D), and pH monitoring (± impedance) off therapy for NERD patients (Grade B).
  o Recommended: pH monitoring for short Barrett’s without erosive esophagitis (Grade B) and barium swallow for suspected hiatal hernia or short esophagus (Grade B).
  o Not Required: PPIs do not need to be discontinued before endoscopy (Grade C).

These guidelines are seminal as they provide clarity on previously controversial aspects of patient selection, reinforcing the need for a thorough preoperative work-up including an upper endoscopy, barium esophagram, pH (Impedance) off PPI if needed and esophageal manometry, thereby establishing clear standards for surgical candidacy. They represent a shift from the use of LA grade C as a benchmark for anti-reflux surgery to LA grade B esophagitis. Strengths include broad international expert participation and consensus methodology. Limitations include that despite a thorough Delphi process, there remains uncertainty on the benefit anti-reflux surgery in several patient populations, such as those with particular symptoms (regurgitation as a main symptom, non-cardiac chest pain, extra-esophageal symptoms, dental erosion), reflux hypersensitivity, those with concomitant IBS and functional dyspepsia, scleroderma, Nutcracker esophagus, or major psychiatry comorbidities. The ICARUS guidelines provide quality evidence for the preoperative work-up of patients prior to ARS and are thus a requisite reading for all surgeons performing foregut surgery.

7. Strate et al. Laparoscopic fundoplication: Nissen versus Toupet two-year outcome of a perspective randomized study of 200 patients regarding preoperative esophageal motility [12].

This study by Strate and colleagues is a two-year follow-up of a prospective randomized study assessing the influence of preoperative esophageal motility on clinical outcomes following Nissen and Toupet fundoplication. Each group was composed of 50 patients who had an esophageal dysmotility disorder and 50 patients who did not. All patients underwent clinical examination, endoscopy, 24 h pH study, and esophageal manometry. The outcomes of interest in this study were success rate (concerning reflux) and complication rate (dysphagia) of a Nissen fundoplication compared to Toupet in the subgroups with and without esophageal motility disorders at two years postoperatively and the impact of preoperative esophageal motility disorders on clinical and manometric outcome.

Key findings:

- 85% satisfaction in both groups
- Higher rate of dysphagia in Nissen group
  o Normal preoperative motility (15 vs 4 patients)
  o Preoperative motility disorder (9 vs 5 patients)
- More endoscopic dilations needed for dysphagia after Nissen.
- Higher reoperation rate following Nissen (15 vs 4 patients, *p* < 0.05)
- Preoperative motility did not predict postoperative dysphagia.
- Similar reflux control between groups

This large, randomized trial is significant as it was the first randomized control trial comparing esophageal motility in patients undergoing partial or total fundoplication. Strengths include its randomized design, standardized technique by experienced surgeons, comprehensive objective testing, and long-term follow-up. While no limitations are highlighted by the authors, two potential limitations which could have impacted the incidence of dysphagia include using a 36 French bougie over which the Nissen fundoplication was created and relatively rapid advancement to a soft diet by postoperative day 3. This RCT provides high-quality evidence demonstrating that both partial and total fundoplication are equally efficacious for acid reflux control, and dysphagia is worse with a total fundoplication regardless of preoperative motility. This study’s findings contribute to surgical guidelines and patient selection criteria for reflux surgery, making it a seminal work with lasting influence.

8. Broeders et al. Systematic review and meta-analysis of laparoscopic Nissen (posterior total) versus Toupet (posterior partial) fundoplication for gastroesophageal reflux disease [13].

This systematic review and meta-analysis compares outcomes of randomized controlled trials examining laparoscopic Nissen fundoplication (LNF) and laparoscopic Toupet fundoplication (LTF) for gastroesophageal reflux disease (GERD). The systematic literature search was carried out in four databases for articles published up to 2009. Seven eligible randomized controlled trials (RCTs) comparing LNF (*n* = 404) with LTF (*n* = 388) were identified and analyzed.

Key findings showed that LNF was associated with

- Significantly higher prevalence of postoperative dysphagia (RR 1.61, *p* = 0.02) and need for postoperative dilatation (RR 2.45, *p* = 0.04)
- Higher reoperation rate (RR 2.19, P = 0.03)
- Higher prevalence of gas-related symptoms including inability to belch (RR 2.04, *p* = 0.009) and bloating (RR 1.58, *p* < 0.001)
- Equivalent reflux control based on acid exposure (RR 1.26, *p* = 0.29), esophagitis (RR 1.20, *p* = 0.40), patient satisfaction, operating time or in-hospital complications

This paper deserves inclusion as a top 10 article because it represents the first comprehensive meta-analysis comparing these two commonly performed anti-reflux procedures, providing level 1a evidence to guide surgical decision-making. The study's strengths include its comprehensive systematic review methodology, selective focus on RCTs, exclusive examination of laparoscopic approach, and analysis of multiple clinically relevant outcomes. Limitations include varying methodological quality of included trials and relatively short follow-up periods of 1–5 years. This paper has significantly impacted surgical practice by providing high-level evidence that partial fundoplication can achieve equivalent reflux control with fewer side effects and lower rates of postoperative dysphagia compared to total fundoplication.

9. Analatos et al. Clinical outcomes of a laparoscopic total vs a 270° posterior partial fundoplication in chronic gastroesophageal reflux disease: a randomized clinical trial [14].

In this article by Analatos and colleagues, the authors report over 15 years of follow-up from a double-blind randomized controlled trial of 456 patients comparing outcomes between total and posterior partial fundoplication for treatment of GERD. The primary outcome was dysphagia scores for solid and liquid food items at > 15 years. Secondary outcomes included quality of life (SF-36), reflux symptoms (GSRS), PPI use, and reoperation rates. Follow-up data were collected between 2019 and 2021 through mailed questionnaires with a 76% response rate (310/407 eligible patients).

Key findings.

- No difference in long-term quality of life between techniques
- Similar reflux control between groups at 15 + years
- The previously described early advantage of less dysphagia with partial fundoplication was no longer evident by long-term follow-up.
- 25–28% PPI usage in both groups at 15 years
- No difference in reoperation rates between groups

This study is a landmark paper as it represents one of the largest randomized trials comparing these techniques with exceptionally long follow-up. The authors concluded that both total and partial posterior fundoplication provide similar long-term control of GERD symptoms and quality of life improvement. The strengths of this paper include the randomized design, large cohort of patients, and exceptionally long follow-up period. The limitations include a proportion of patients lost to follow-up, though relatively small, and lack of objective testing (pH studies) at long-term follow-up. This study represents a seminal paper in the field due to its high-quality methodology and uniquely long follow-up period showing equivalent long-term outcomes between the techniques despite some early advantages of partial fundoplication for postoperative dysphagia.

10. Nissen. Gastropexy and “Fundoplication” in Surgical Treatment of Hiatal Hernia [15].

This landmark 1961 paper by Nissen describes the technical principles and early outcomes of the fundoplication procedure that would later become the standard anti-reflux operation, thereby establishing the fundamental principles and technical considerations that remain relevant today.

In this paper, Nissen describes the development and early outcomes of gastropexy and fundoplication for treating hiatal hernias. The gastropexy technique was first used as an emergency solution for an elderly patient with incarcerated paraesophageal hernia, while the fundoplication technique evolved following the repair of a perforated ulcer of the gastric cardia. In this case, he describes, “to reinforce the sutures connecting the esophageal stump and the stomach, the latter was mobilized, and the distal segment of the esophagus implanted in it in much the same manner as the rubber tube in Witzel’s gastrostomy.” He later observed that this technique resulted in the long-term prevention of reflux leading to the adoption of this practice in combination with gastropexy. The fundoplication technique is described as creating an abdominal incision, mobilizing the distal part of the esophagus 6 cm into the abdominal cavity, wrapping of the fundus around the esophagus and fixating the gastric folds with sutures. In a series of 96 patients who underwent fundoplication plus gastropexy, Nissen reports 88% of patients demonstrated radiographic resolution of their hiatal hernia over an observation period of 3 months to 3.5 years. Complications included one mortality from peritonitis, two cases of wrap failure due to suture separation, and some cases of temporary postoperative stenosis.

The main strength of this paper is that it was the first description of the fundoplication technique with technical considerations based on actual surgical experience. Furthermore, Nissen provided outcomes and insights on the early successes and challenges of this technique. The limitations are inherent to the descriptive nature of the paper, including its small sample size with limited long-term follow-up and no comparator arm.

In summary, Nissen concluded that fundoplication represents a simple and effective surgical approach for treating hiatal hernia and reflux. In the decades following this paper, the Nissen fundoplication became the gold standard operation for GERD and remains one of the most commonly performed anti-reflux procedures worldwide. This paper represents a foundational contribution to anti-reflux surgery, and the technical principles described continue to guide surgical decision-making in modern practice. Its inclusion in the top 10 seminal articles reflects its enduring influence on the field of foregut surgery.

## Conclusions

The seminal articles presented here represent key foundational knowledge required for understanding and performing laparoscopic fundoplication. From Nissen’s original description to modern randomized controlled trials, these papers establish the technical principles, patient selection criteria, and evidence supporting fundoplication as safe, effective, and durable treatment option for gastroesophageal reflux disease when appropriate technical principles are followed. Key papers include clinical practice guidelines for patient selection and technical considerations and randomized controlled trials comparing different surgical techniques and surgical versus medical therapy. The knowledge derived from these papers serves as both a practical guide for surgeons learning or refining the procedure, as well as a framework for understanding the scientific principles that underpin successful anti-reflux surgery. These findings have directly informed modern practices and continue to guide ongoing refinements in technique and discovery. As new evidence emerges regarding technical modifications, patient selection, and outcomes, our understanding will evolve. However, these seminal papers provide the essential foundation for surgeons to develop expertise in laparoscopic fundoplication within the SAGES Master’s program framework.

## Supplementary Information

Below is the link to the electronic supplementary material.

Supplementary file1 (DOCX 13 KB)
