# Supplementary material for: The SAGES masters program: top 10 seminal articles for laparoscopic fundoplication
Source: Surg Endosc. 2025 Sep 8;39(10):6301–9. doi: 10.1007/s00464-025-12141-1 (PMC12500779; doi:10.1007/s00464-025-12141-1)
Supplement: Supplementary file 1 — Supplementary file1 (DOCX 13 KB) [file 464_2025_12141_MOESM1_ESM.docx]

**Supplementary 1. Librarian Search Terms**

**Databases:** Web of Science, Google Scholar

**Article type**: clinical trial, meta-analysis, systematic review, guidelines

**Competency Search Terms -** Fundoplication/Nissen

**Focused Statement:** Laparoscopic fundoplication of Foregut

**Procedure/Intervention:** Laparoscopic Nissen fundoplication, Laparoscopic Toupet fundoplication, partial fundoplication, total fundoplication, Nissen, Toupet. Robotic fundoplication, robotic Nissen, Robotic Toupet, laparoscopic Dor, robotic Dor

**Problem/Disease:** gastroesophageal reflux disease, esophagitis, GERD, laryngopharyngeal reflux, LPR

**Additional terms:** Foregut, Gastroesophageal reflux Disease, GERD, sliding hiatal hernia, ineffective esophageal motility, acid reflux, heartburn, regurgitation.
**Dates:** 2000- Present

**Exclude:** animal studies, studies on patients <18 years old, Redo repair, Revisional repair, Recurrent hernia repair, Recurrent hernia, Paraesophageal hernia, Recurrent paraesophageal hernia
